# Analysis of Filler Metals Influence on Quality of Hard-Faced Surfaces of Gears Based on Tests in Experimental and Operating Conditions

**DOI:** 10.3390/ma15217795

**Published:** 2022-11-04

**Authors:** Svetislav Marković, Vukić Lazić, Dušan Arsić, Ružica R. Nikolić, Djordje Ivković, Robert Ulewicz, Otakar Bokuvka

**Affiliations:** 1 Faculty of Technical Sciences Čačak, University of Kragujevac, 32000 Čačak, Serbia; 2 Faculty of Engineering, University of Kragujevac, 34000 Kragujevac, Serbia; 3 Research Centre, University of Žilina, 010 26 Žilina, Slovakia; 4 Department of Production Engineering and Safety, University of Technology, 42201 Czestochowa, Poland; 5 Faculty of Mechanical Engineering, University of Žilina, 010 26 Žilina, Slovakia

**Keywords:** hard-facing, gears, hardness, microstructure, tribological tests, wear

## Abstract

Hard-facing as a type of the coating depositing is increasingly used today. Physical-chemical-metallurgical characteristics of contact layers in tribo-mechanical systems depend on the operating conditions and the conditions under which the work surfaces were created. That is the reason the influence of the processing procedures and regime, used in the contact surfaces formation, on development of the wear process of contact elements, is being considered ever more. To determine the influence of the hard-facing technology on characteristics of the gears’ working surfaces, the experimental investigations were performed on samples hard-faced on the steel for cementation, by varying the filler metals (FM) and the hard-facing regimes. The samples tested were hard-faced by five “hard” and three “soft” filler metals. Experimental investigations included measuring the hard-faced layers’ hardness and determination of their microstructure, as well as the wear resistance in the laboratory conditions, on tribometer and on a specially designed device for tests in the real operating conditions of gears. The wear intensity was monitored by the wear trace’s width in the laboratory conditions and by the share of the teeth surfaces affected by the destructive pitting in the operating conditions. The results obtained were compared to results of the base metal (BM) tests, which provided the certain conclusions on which filler metal and which welding procedure are the optimal ones for regeneration of the worn teeth surfaces.

## 1. Introduction

All the replaceable elements and other parts, installed in various subassemblies and assemblies of machine tools, construction and agricultural machines, ironworks rollers, presses and forging hammers, and road and rail vehicles, can, as a rule, be regenerated after normal or accidental wear. Numerous welding and thermal metallization methods are available for this. Today, it is considered that for over half of the replaceable parts, it is economically justified to be repair and re-install them in appropriate machines and devices. However, despite the economic justification, one has to take into account the unfavorable structural changes, residual stresses, cracks and other consequences that follow reparatory hard-facing [1]. From the view-point of operating characteristics, the most important are hardness and structure of the hard-faced layers; however, some other properties are important, as well, like the strength of adhesion between the base metal (BM) and coating [2], residual thermal and structural stresses, [3] and sometimes the resistance to wear and corrosion [4]. For the sake of reducing the replacement’s costs, namely increasing the service life of certain vital parts and assemblies of machines and devices, it is of the utmost importance to choose the adequate welding procedure, the filler metal (FM), and to prescribe the optimal technology of hard-facing. The point of optimization is to choose such procedures, filler metals, welding parameters and heat treatment to obtain the best output characteristics of the weld. The established general procedure for hard-facing of one type of parts can be applied to repair of parts made of other types of materials and different geometric shapes [5].

Thus, the hard-facing today represents very often applied technology for regeneration of the damaged work parts. Application of hard-facing has full techno-economic justification and it offers a number of advantages, such as shortening the time needed for repairing the damaged parts, savings in the storage room, money, etc. [6]. In addition, necessity of hard-facing also implies creating a company’s own technological department to be responsible for it, engaging the expert staff, equipment, and etc., which makes the company less dependent on external market influences.

Damages to the parts in contact the most frequently appear as a consequence of tribological influences [7,8,9]. However, nowadays, those can be eliminated by hard-facing, due to the fast technological development, while taking into account the base metal [9,10]. Additionally, by application of hard-facing, one can expect to achieve the satisfactory quality of the regenerated parts.

Experience of present authors in this area is great and long, so the aforementioned benefits of hard-facing are proven through a number of successfully applied repairs in various parts of the machinery industry. Some examples are the regeneration of forging tools and parts of the forging industry [11,12,13,14,15], where it was pointed out that the function of the damaged part can be restored very quickly and thus achieve savings in terms of downtime, savings on material and energy, employment etc. Similar principles are emphasized in papers [16,17], on examples of construction machinery parts, in works [18,19] on examples of heavy mining equipment parts, as well as responsible parts of machine constructions, such as gears, whether they are the author’s own research [20,21,22] or research by other authors [23,24,25,26,27,28,29]. In all the mentioned research, it has been shown that hard-facing achieved at least the same characteristics of the applied welds in comparison to the basic materials, while it is often the case that their resistance is even higher. This indicates the possibility of extending the service life of welded parts with respect to the new ones.

The hard-faced surfaces of the working parts are in the operating conditions, in the majority of cases, subjected to tribological influences, which would inevitably lead to their repeated degradation [30,31]. That is why it is important to be aware of the degree of wear of the hard-faced layers, with respect to the base metal, as well as to know all the phenomena that can affect the process of loss of serviceability of machine systems in operating conditions [32,33]. Therefore, for securing the necessary level of quality and reliability of machines’ operation, it is necessary to analyze all the phenomena that are accompanying the process of manufacturing and exploitation of the machine systems and their parts.

The degree of wear can be monitored via the certain parameters, which are pointing to loss of the working ability of products and value of the working resources. The type of wear is usually determined by the operating conditions and regime of a machine, value and shape of the clearance between the assemblies, etc. To a certain extent, this also affects the rigidity of the system and the magnitude of the specific pressures between the contact surfaces. All of these greatly affect the intensity of wear in a certain period of work. At the same time, the intensity of wear of the work surfaces depends on the macro- and micro-geometry of contact surfaces, micro-hardness, structure, and stress state of the surface layers, formed in the manufacturing process.

This paper presents the detailed analysis of tribological characteristics of various filler metals by application of different welding procedures (MMAW—Manual Metal Arc Welding and GTAW—Gas Metal Arc Welding). The steel for cementation was used as a base metal, which is otherwise used for gears’ manufacturing. The tribological test implied the block-on-disc contact and monitoring of the wear intensity of the hard-faced layers. The testing was conducted in the limit lubrication conditions. Besides the tribological tests, an analysis of the microstructure was performed, as well as the hardness measurements, which is a very important indicator of the material’s wear resistance. The second part of tests consisted of the wear resistance investigation of the hard-faced layers’ surfaces on a specially constructed device, which was simulating the operating conditions of the gears.

## 2. Preparation of Samples for Experimental Investigations of the Hard-Faced Surfaces

**Figure 1:**
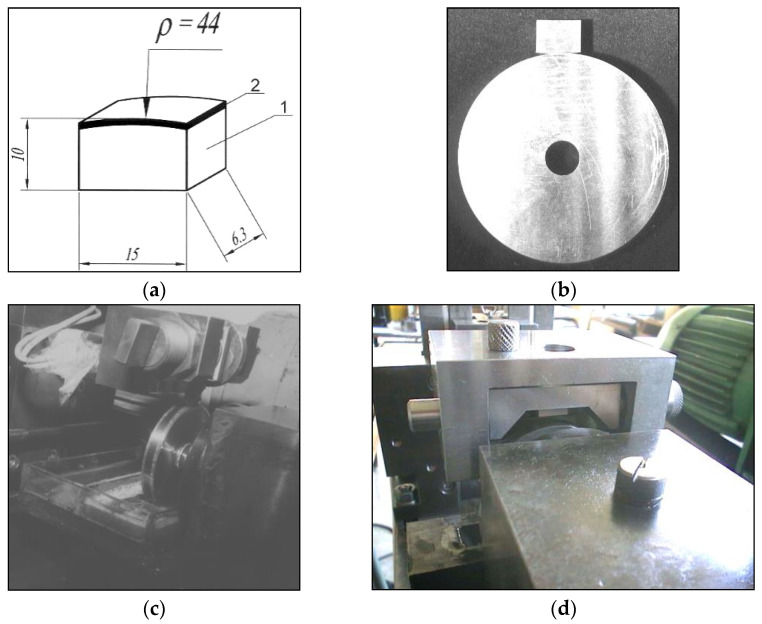
Tribological tests illustration: (**a**) Tribometer samples tested on tribometer (1—the base metal, 2—hard-faced layer, ρ—tooth curvature radius); (**b**) Appearance of the disc and block prepared for the test; (**c**) Tribometer TPD-3 with a disc submerged into oil and block above; (**d**) Block fixed in the holder before establishing the contact with the rotating disc.

The test samples were made of the cementation steel 20MnCr5, the chemical composition of which is given in Table 1. Those samples were prepared in such a way that they could simulate operation of the meshed teeth of the concrete gears, in conditions set in the device for the exploitation investigation, Figure 1.

Discs for the tribological tests, of dimensions ∅60 × 10 mm, were made of the same steel. After the machining on a lathe, they were gas cemented, quenched, tempered, and then ground. Since all the discs were made from the same material (one batch), treated by the same machining regime, as well as cemented and heat treated, they possessed almost identical surface hardness, which was within range from 56 to 56.2 HRC. Tribological test was conducted according to standard ASTM G99-17.

For the testing purposes, a large number of samples (blocks) were hard-faced with different types of filler metals, welding procedures, and were subsequently heat treated. A total of nine types of test blocks have been prepared, which can be divided into three groups, according to the method of construction:

- In the first group, the samples were welded with “hard” filler materials. Five different types of samples were made, and the preparation technology is shown in Table 2. The preheating temperature was 230 °C and the electrodes were dried for 4 h at a temperature of 350 °C. After the hard-facing, the samples were returned to the oven, where they were heated to the preheating temperature and cooled to room temperature.
- The second group consisted of samples hard-faced with the “soft” filler metal, which were then cemented, quenched, and tempered. There were three types of those samples, hard-faced by technology presented in Table 3. The preheating and welding procedures were identical for all the samples of this group, while the differences were that some samples were soft annealed, then gas cemented, quenched, and tempered.
- In the third group were samples that were not hard-faced, but gas cemented, quenched, and soft annealed. These samples were simulating the operation of the newly manufactured, not-regenerated gear teeth. The exception is hard-facing of samples marked by ordinal number 1, in which the intermediate layer was first welded with the Inox 18/8/6 electrode and, after the slag removal and cleaning, with the E DUR 600 electrode.

After the hard-facing and heat treatment, the samples’ deposited surfaces were ground on the machine for circumference grinding. The samples round radius (Figure 1) corresponds to the calculated value of the mean radius of the gear teeth being regenerated:

$$\rho =R_{1}\cdot \sin\alpha =129\cdot \sin20{}^{\circ}≅44\;\mathrm{mm}.$$

Notation and dimensions of the used filler metals (diameter D) and power intensity (J) of the hard-facing process are given in Table 4.

**Table 2:** Preparation technology of samples hard-faced by the “hard” filler metal.

Sample Number	Base Metal	Pre Hard-Facing Heat Treatment	Hard-Facing		Post Hard-Facing Heat Treatment
			Filler Metal	Welding Procedure According to AWS	
1	20MnCr5	Preheating	Inox 18/8/6 + EDUR 600	MMAW (111)	Low-temperature tempering
2			Castolin 2		
3			DUR 600-IG	GTAW (TIG) (141)	
4			UTP 670	MMAW (111)	
5			Tooldur		

**Table 3:** Preparation technology of samples hard-faced by the “soft” filler metal.

Sample Number	Base Metal	Pre Hard-Facing Heat Treatment	Hard-Facing		Post Hard-Facing Heat Treatment
			Electrode	Welding Procedure According to AWS	
6	20MnCr5	Preheating	EVBCrMo	MMAW (111)	Soft annealing + cementation + quenching + tempering
7			EVB2CrMo		
8			Phönix 120K/E		

**Table 4:** Characteristics of the applied filler metals.

Sample No.	Designation According to			Manufacturer	Welding Procedure (AWS)	D (mm)	J (A)
	EN	AWS	DIN 8555				
1	E-6-UM-55G	/	EDUR 600	Jesenice (Slovenia)	MMAW (111)	2.5	70
2	/	Castolin 2	/	Castoline Eutectic (Switzerland)		3.25	92
3	MSG-6-GZ-60	/	DUR 600-IG	BÖHLER (Germany)	GTAW (TIG) (141)	1.2	76
4	E-6-60-UM	/	UTP 670	UTP (Germany)	MMAW (111)	3.25	90
5	E-4-UM-60-65-S	EFe5-B	Tooldur	Jesenice (Slovenia)		2.5	80
6	ECrMo1B26	E8018-B2	EVBCrMo	Jesenice (Slovenia)		2.5	75
7	ECrMo2B26	E9018-B3	EVB2CrMo	Jesenice (Slovenia)		2.5	75
8	/	/	Phönix 120 K/E 425 B/E7018-1	Tüssen (Germany)		2.5	72
9	E18.8.Mn6B20+	E307-15	Inox 18/8/6	Jesenice (Slovenia)		2.5	70

The technological processes of sample preparation (gear regeneration) by reparative hard-facing with “hard” filler metals are different from the three following procedures.

**Figure 2:**
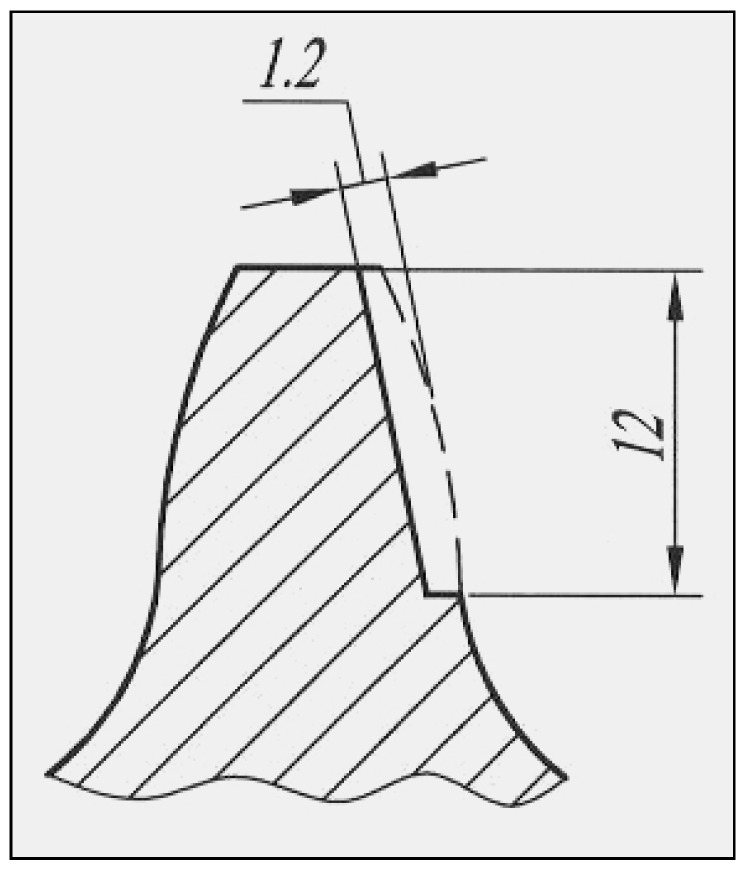
Schematic presentation of the tooth prepared for hard-facing.

The regeneration process of the gear sectors hard-faced in two layers (Inox 18/8/6 + EDur 600) consists of the following operations:

- removal of the cemented layer from the active working surfaces of the teeth by grinding on a sharpener (in practice that corresponds to removing the damaged layer of the side of the tooth) to a depth of 1.5 + 0.2 mm and 12 mm along the height of the tooth (Figure 2),
- preheating the gear to temperature T = 230 °C and keeping it at that temperature for t = 2 h,
- drying of basic electrodes at temperature T = 350 °C for t = 4 h,
- hard-facing of prepared surfaces with filler metal Inox 18/8/6 (coated electrodes ϕ 2.5 mm) with the electric current of strength I = 90 A,
- returning the welded gear to the furnace and slowly cooling it together with the furnace,
- milling of welded surfaces to a depth of 1.2 mm on a universal milling machine,
- preheating the gear to temperature T = 230 °C and keeping it at that temperature for t = 2 h,
- drying of electrodes at temperature T = 350 °C for t = 4 h,
- hard-facing of prepared surfaces with filler metal EDur 600 (with coated electrodes ϕ 2.5 mm), electric current of strength I = 70 A,
- returning the welded gear to the furnace and slowly cooling it together with the furnace,
- grinding of welded teeth on the “Niles” gear grinding machine.

For the hard-facing of teeth with the following three types of filler metals (Castolin 2, DUR 600-IG and UTP 670), the regeneration is somewhat simpler and faster. The technological processes of regeneration of the gear sectors hard-faced with these filler metals consist of the following operations:

- removal of the cemented layer from the active working surfaces of the teeth by grinding on a sharpener (in practice that corresponds to removing the damaged layer of the side of the tooth) to a depth of 1.2 + 0.2 mm and 12 mm along the height of the tooth,
- preheating the gear to a temperature of T = 230 °C and keeping it at the reached temperature for t = 2 h,
- drying of electrodes at temperature T = 350 °C for t = 4 h,
- MMAW hard-facing of prepared surfaces with filler metals Castolin 2 (coated electrodes ϕ 3.25 mm, I = 92 A), DUR 600-IG (TIG process, in argon protection and wire ϕ 1.2 mm), and UTP 670 (coated electrodes ϕ 3.25 mm, I = 90 A),
- returning the welded gears to the furnace and slowly cooling together with the furnace,
- grinding of welded teeth on the “Niles” gear grinding machine.

**Figure 3:**
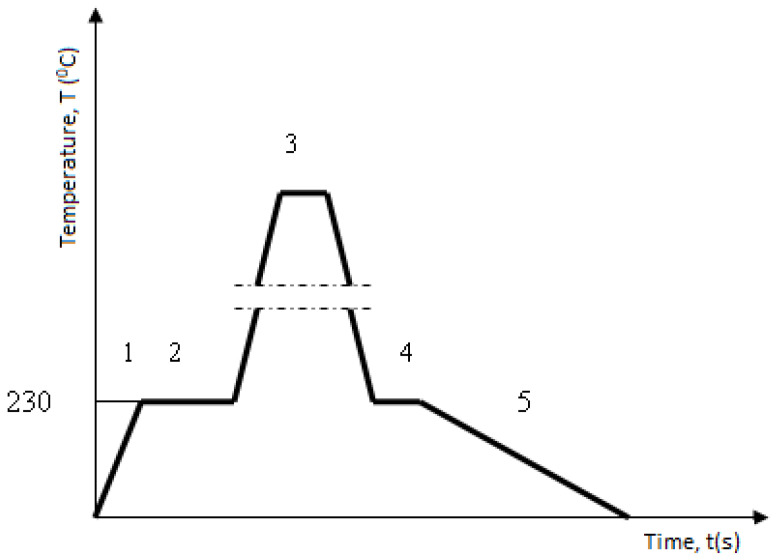
Diagram of the heat treatment regime during the hard-facing: 1—slow heating in the oven; 2—holding at the preheating temperature (T = 230 °C, t = 2 h); 3—hard-facing; 4—returning to the furnace (T = 230 °C, t = 1 h); 5—slow cooling together with the oven.

In the case of tooth regeneration by hard-facing with “soft” filler metals (EVBCrMo and EVB2CrMo), the technological regeneration procedure was significantly different from the previously described ones. It consists of the following operations (Figure 3):

- soft annealing. The soft annealing operation is necessary to reduce the surface hardness of the working surfaces of the teeth and to enable preparation by milling. Annealing was performed in a vacuum by heating to a temperature of T = 680 °C, at a rate of 10 °C/min. Then, the gears were kept at that temperature for 4 h, then cooled at a rate of 20 °C/min.

- removal of the cemented layer from the active working surfaces of the teeth by milling on a universal milling machine (in practice that corresponds to the removal of the damaged layer of the side of the tooth) to a depth of 1.2 + 0.2 mm and 12 mm along the height of the tooth,
- preheating the gear to a temperature of T = 230 °C and keeping it at that temperature for t = 2 h,
- drying of electrodes at temperature T = 350 °C for t = 4 h,
- MMAW hard-facing of prepared surfaces with filler metals EVBCrMo (coated electrodes ϕ 2.5 mm) and EVB2CrMo (coated electrodes ϕ 2.5 mm),
- high tempering in the furnace,
- oversize milling (with grinding attachment) on a milling machine for manufacturing gears using the Pfauter method,
- soft annealing,
- cementation in a mixture of CO_2_ + CO to a depth of 1 ± 0.1 mm with cooling in the pit,
- quenching and tempering in the salt solution,
- grinding of welded teeth on the “Niles” gear grinding machine.

The newly made gears and gears welded with “soft” filler metals were cemented in an active gas atmosphere under the following conditions:

- cementation furnace—multipurpose chamber furnace with protective gas type VKEs-3 (“Ajchelin”—CER),
- carbonizing gas—endogas with a carbon potential of 0.35% C with the addition of about 4% propane,
- gas mixture dosing regulation—automatic,
- measurement and regulation of gas atmosphere composition—oxygen probe type OCC 2000 (“Progress-electronic”),
- regulation of the total cycle (composition, temperature, time)—automatically using built-in industrial controllers.
- Quenching was performed directly from the cementation temperature.
- Grinding of all gears (both newly made and regenerated) was carried out on a “Niles” gear grinding machine, with the following working characteristics:
- number of revolutions of the electric motor: n = 1400 min^−1^,
- tool speed (auxiliary movement): s = 960 mm/min,
- peripheral speed of the workpiece: v = 510 mm/min.

During the grinding, intensive cooling is provided to prevent structural and mechanical changes on the working surfaces of the teeth.

**Figure 4:**
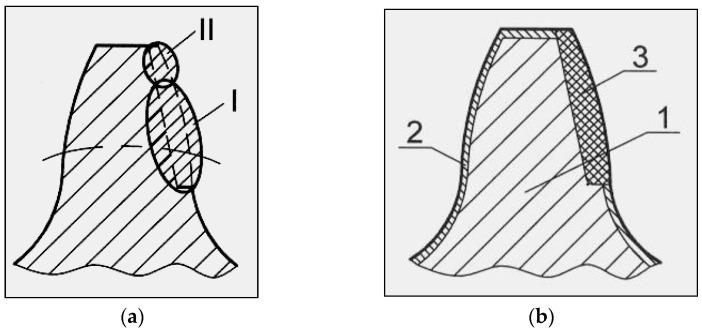
(**a**) Appearance of the tooth hard-faced in two passes; (**b**) Appearance of the tooth cross-section after the reparative hard-facing and machining (1—the tooth core; 2—the cemented layer; 3—the hard-faced part of the tooth flank).

The hard-facing of all teeth was performed in two passes. In the first pass, the bottom of the tooth and part of the head (I) were hard-faced and, after removing the slag, the remaining part of the head (II) was welded, Figure 4a. “Fronius” brand welding machine was used to perform the welding operations.

When hard-facing the teeth, it was noted that the thickness of the welds must be increased by the size of the additions for the planned subsequent machining.

## 3. Microstructure Investigation

Metallographic investigations were conducted on the quantitative optical metallographic microscope of the “Polyvar-Met” type (“Reichert–Jung”) at magnifications of 20 to 2000 times. The microstructural analysis was done on the “Leica Q500MC” device.

**Figure 5:**
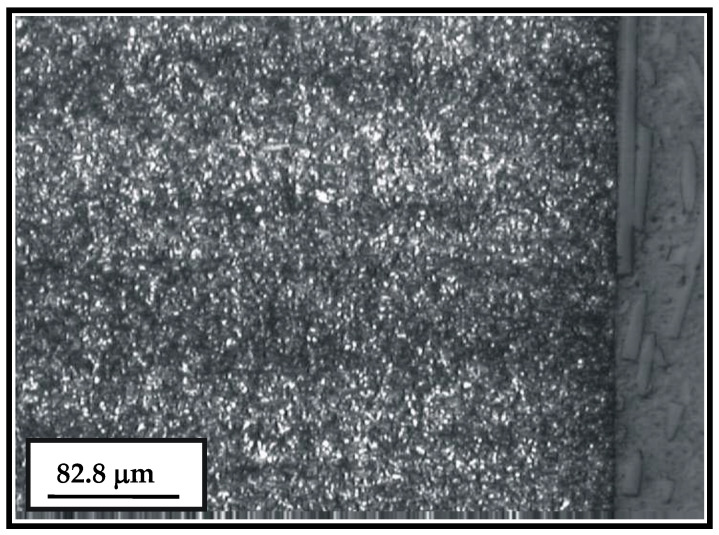
Microstructure of the cemented layer.

**Figure 6:**
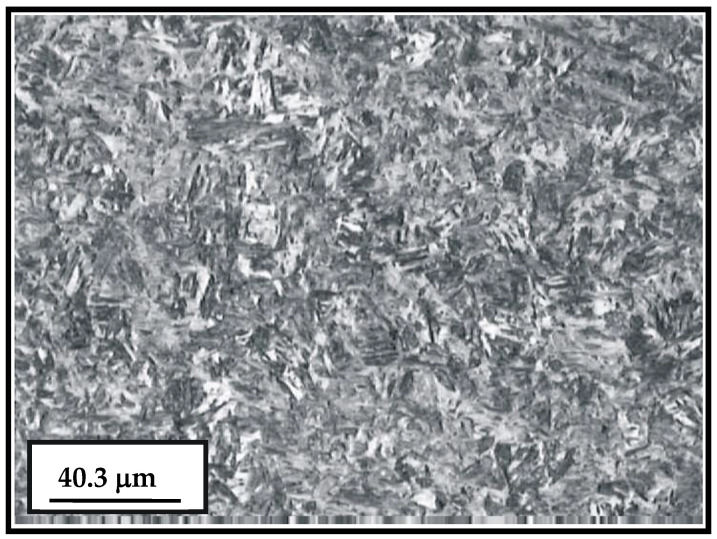
Structure of the cemented sample bulk.

The basic microconstituent of the cemented layer is the tempered martensite (dark areas in Figure 5). The microstructure of the surface and sub-surface (cemented layer and the bulk) of the newly manufactured sample is shown in Figure 6.

The smaller share of residual austenite can be noticed in the microstructure. In the surface zone, depths of up to 0.2 to 0.3 mm are present the individual larger carbides of the alloying elements. Based on the martensite needles appearance, it can be concluded that the microstructure is fine-grained, which points to the fact that the heat treatment regime was appropriately executed. In Figure 6, it can also be noticed that the microstructure contains larger needles of the transient structures (bainite) and the ferrite areas.

**Figure 7:**
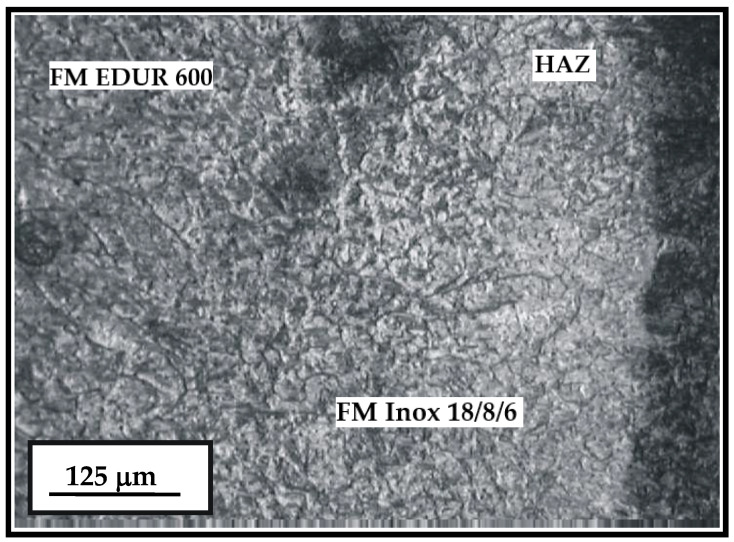
Microstructure of the HAZ and the hard-faced layer with the two filler metals—Inox 18/8/6 + EDUR 600; EDUR 600—left; Inox 18/8/6—middle; HAZ—right.

The microstructure of the hard-faced layer deposited with the two different filler metals (Inox 18/8/6 and EDUR 600) is shown in Figure 7. On the left-hand side of the figure, one can notice the bar-like dendritic structure of the hard-faced layer deposited by the “hard” electrode EDUR 600. In the central portion of the figure is the hard-faced layer deposited by the electrode Inox 18/8/6 with the less prominent expressed dendritic structure. The joining line of the FM Inox 18/8/6 and the heat affected zone (HAZ) of the base metal is, relatively, prominent and very uneven.

**Figure 8:**
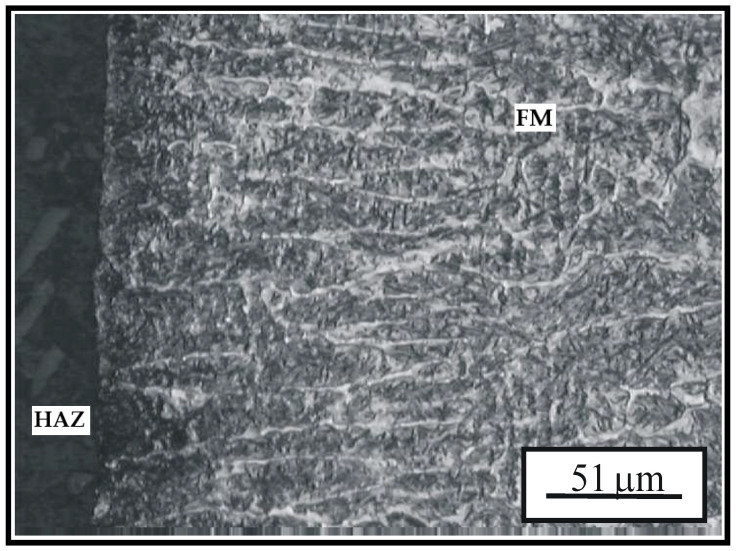
Microstructure of the hard-faced layer executed by the FM Castolin 2.

Figure 8 presents a microstructure of the hard-faced layer executed by the FM Castolin 2. According to Figure 8, the layer hard-faced by the FM Castolin 2 is characterized by prominent dendritic structure. The average width of dendritic needles is 10–15 μm, which is very favorable. However, the dendrites are highly inhomogeneous, which is shown by the different etching degree of their surfaces. In the layer, hard-faced by Castolin 2, the carbide phase is present, excreted mainly at the dendritic boundaries. The growth direction of dendrites is mainly perpendicular to the sample’s surface.

**Figure 9:**
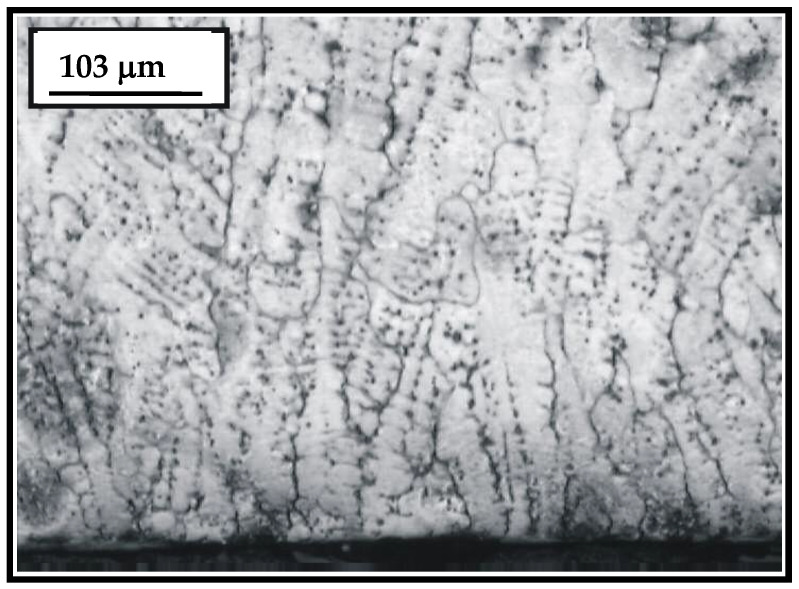
Elongated dendritic structure of the hard-faced layer executed by the GTAW welding procedure and filler metal DUR 600-IG.

Figure 9 shows the elongated dendritic structure of the hard-faced layer executed by the GTAW welding procedure and filler metal DUR 600-IG that extends all the way to the surface of the hard-faced sample. The registered dendrites have the growth direction perpendicular to the working surface; the dark spots on their edges are mainly excreted impurities, carbide phase and alike.

**Figure 10:**
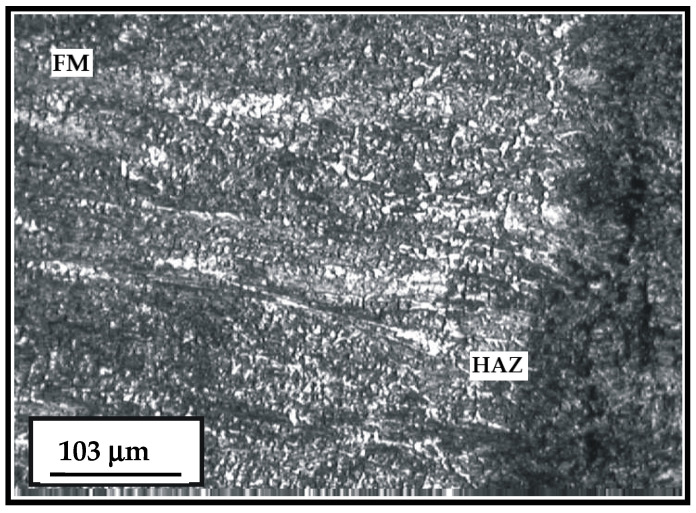
Microstructure of the hard-faced layer executed by FM UTP 670 (light areas) and HAZ (dark area).

The sample hard-faced with the FM UTP 670 has the medium to large-grained structure, with individual dendrites width of 30 to 80 μm. The large share of excreted carbides along the grain boundaries is noticed in the structure (light areas in Figure 10), however the largest portion is within the metal grains.

**Figure 11:**
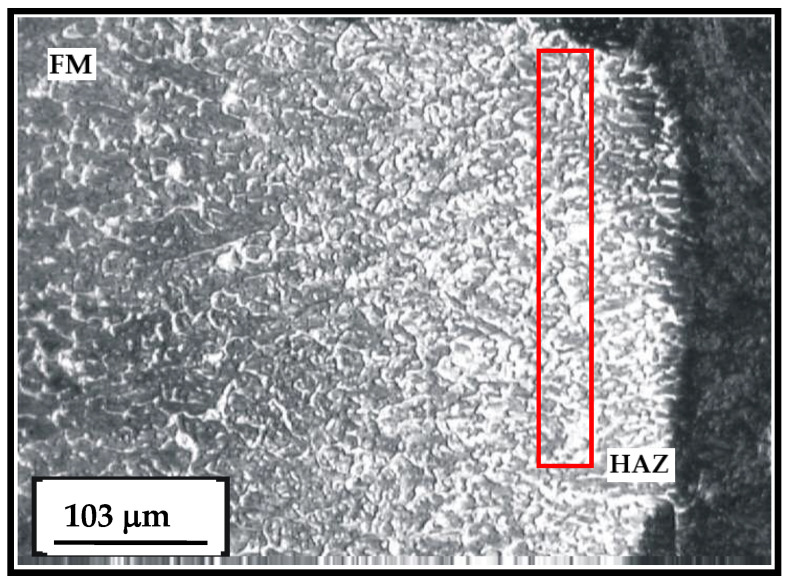
Microstructure of the hard-faced layer executed by the FM Tooldur (light area) and HAZ (dark area); the red-framed area is the zone of the largest portion of the carbide phase.

The hard-faced layer obtained by the FM Tooldur is characterized by prominent dendritic structure. From comparison of microstructures of all the hard-faced layers, one can conclude that in this structure, the largest share of the carbide phase is present (Figure 11). The largest portion of the carbide phase is in the area immediately next to the HAZ, where it is excreted both on the grain boundaries and within the grains. The smaller share of the carbide phase is present in other zones of the hard-faced layer and at the surface, where this phase is mainly excreted along the edges of dendrites and grain boundaries.

**Figure 12:**
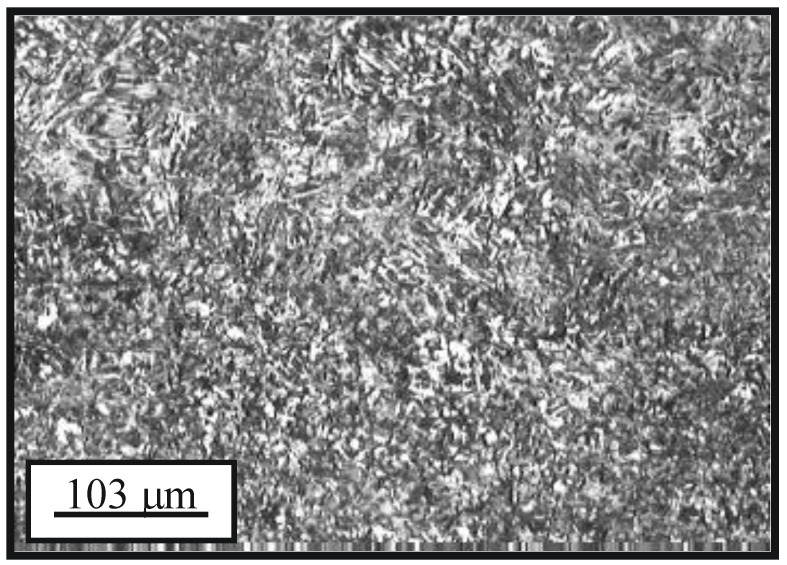
Microstructure of transition between the cemented layer and transient layer in the hard-faced layer executed by the FM EVBCrMo, chemically and heat treated.

Figure 12 shows the microstructure in the transition zone between the cemented layer (fine-grained structure) and the transient cementation zone, which is present in the hard-faced layer (executed by the FM EVBCrMo), as well. The basic microconstituent in this structure is the tempered martensite with the smaller share of the carbide phase and residual austenite. The size of the martensitic needles is mainly fine grained, which points to the fact that the soft annealing process after the hard-facing and subsequent chemical and heat treatment processes were correctly executed.

**Figure 13:**
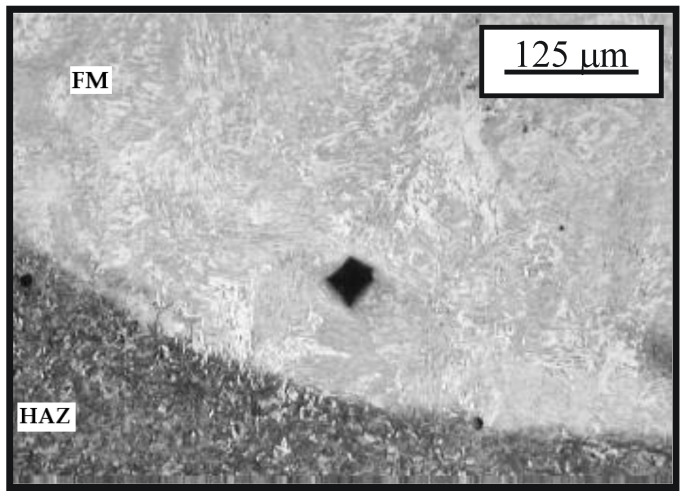
Microstructure of the transition zone between the hard-faced layer executed by the FM EVB2CrMo (light area) and the base metal (HAZ); state: hard-faced + soft-annealed + cemented + heat treated.

The microstructure of the cemented layer in the hard-faced layer executed by the FM EVB2CrMo is shown in Figure 13 [21]. The first cemented and then heat-treated layer is characterized by somewhat greater inhomogeneity with respect to the layer hard-faced by FM EVBCrMo. The basic microconstituent in the hard-faced layer zone is the tempered martensite. The hard-faced layer is the light area in the figure, while the base metal (namely the HAZ) is the dark area. Based on difference in the etching intensity of the base metal and the hard-faced layer, one can conclude that there is a more prominent difference in their chemical compositions.

**Figure 14:**
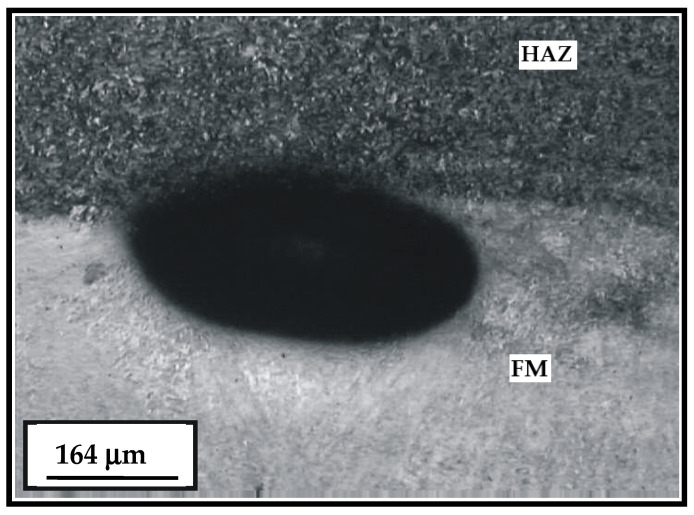
Microstructure of the hard-faced layer executed by the FM Phönix 120 K/E with appearance of the gas bubble in the HAZ.

The hard-faced layer microstructure obtained by the filler metal Phönix 120 K/E is noticeably medium to fine-grained (Figure 14). Based on the degree of etching intensity, one can establish the difference in the chemical composition between the base and filler metal. An example of the individual gas bubble at the joining line BM-FM is also shown.

## 4. Investigations of Hardness and Microhardness

**Figure 15:**
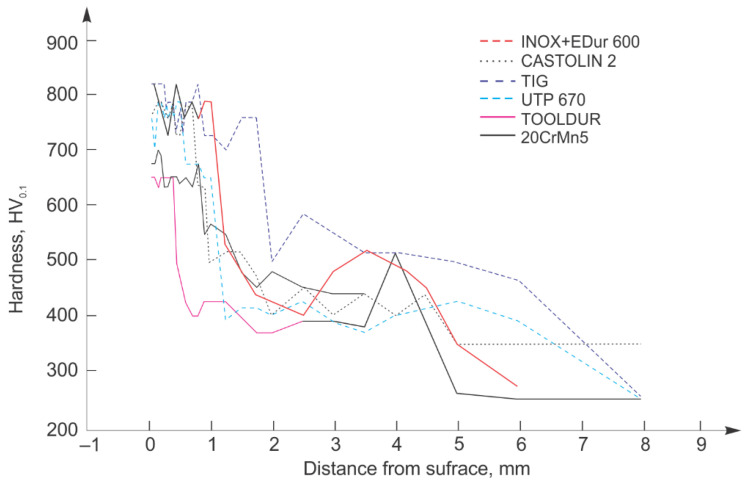
Hardness distribution diagram of samples hard-faced with “hard” filler metals.

Very important indicator of the wear resistance of the regenerated samples is the working surfaces hardness. Besides the surface and macrohardness, it also is necessary to check their microhardness along the cross-section. Within the experimental part, the surface hardness was measured by the Rockwell method (Figure 15) at five points on the “Leitz Wetzlar” device. Average values are entered into Table 5. One can notice that the newly made samples possess the highest hardness; however, the most important conclusion is that the surface hardness of all the hard-faced layers is within the required limits (58 ± 3 HRC).

Microhardness was measured on the same device by the Vickers (HV0,1) method with indentation force of 1 N and indentation time of 15 s. Results of the microhardness measurements of the samples hard-faced by the “hard” filler metals, and the newly made samples are shown in Figure 15.

**Figure 16:**
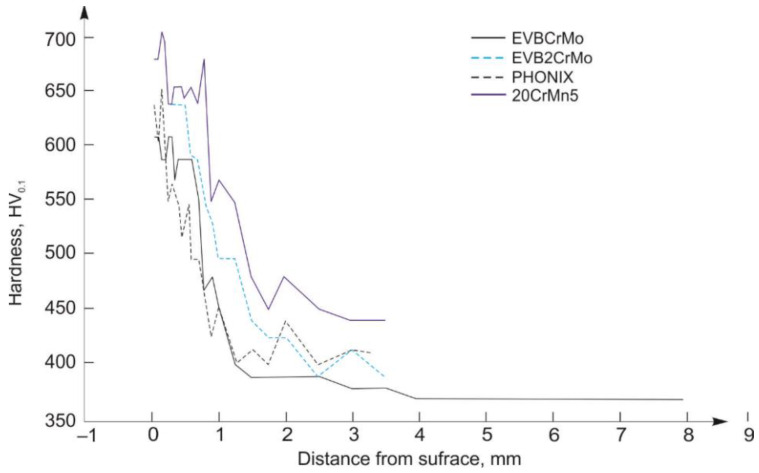
Hardness distribution diagram of samples hard-faced with “soft” filler metals.

Microhardness of samples hard-faced by the “soft” filler metals, cemented and heat treated, are shown on a diagram in Figure 16.

**Table 5:** Surface hardness and macrohardness of the hard-faced and newly made samples.

Sample No.	Regeneration Method	Hardness HRC
1.	MMAW h-f FM Inox 18/8/6 + EDUR 600	57.5
2.	MMAW h-f FM Castolin 2	58
3.	MMAW h-f TIG procedure FM DUR 600-IG	56.5
4.	MMAW h-f FM UTP 670	55.5
5.	MMAW h-f FM Tooldur	56
6.	MMAW h-f FM EVBCrMo + C + HT	57
7.	MMAW h-f FM EVB2CrMo + C + HT	56
8.	MMAW h-f FM Phönix 120 K/E 425 B/E7018-1+ C + HT	56.5
9.	Newly made (20MnCr5 + C + HT)	59

h-f—hard-faced; C—cemented; HT—heat treated.

From comparison of the microhardness distribution of the newly made samples and those hard-faced by the “soft” FM and then cemented and heat treated, one can notice that the microhardness of the latter samples is lower than of the former ones. The cause for this phenomenon can be found in the fact that all the samples were treated according to the chemical–heat treatment prescribed for cemented layers on the 20MnCr5 steel, which was previously hot, plastically deformed and softly annealed.

The subject of selecting the optimal regime for the chemical-heat treatment can be a topic of separate research.

From the aspect of the microhardness distribution of the “softly” hard-faced samples, hard-faced layers executed with the EVB2CrMo FM possess the microhardness values very close to those of the newly made samples. For the hard-faced layers executed with the EVBCrMo FM, the hardness distribution is somewhat less favorable, while the hard-faced layers executed with the Phönix 120 K/E 425 B/E7018-1 FM possess the worst microhardness distribution.

Comparing the microhardness distribution of the newly made and “hard” hard-faced layers, one can notice that the latter mainly possess prominently higher microhardness of the subsurface layers with respect to the cemented layers, with exception of the hard-faced layers executed with the Tooldur FM. 

## 5. Tribological Investigations

Tribometer was used for the tribological tests, where the blocks were made from samples hard-faced by the selected filler metal coupled with a disc made of 20MnCr5 steel, cemented, and quenched. The linear contact was assumed between the disc and a block. To make the model investigations as close as possible to the experimental ones, the blocks were made with the rounding radius that corresponds the Wentzler teeth rounding radius ρ = 44 mm, while the disc diameter was ∅60 mm. Those radii were not arbitrarily selected; they correspond to the teeth curvature radii during the certain points of contact (meshing with other teeth), namely at the beginning of the contact and at the exit of it, when the sliding is the biggest.

Besides that, the roughness parameters of each block and disc were measured, prior to and after the tests, which enabled monitoring their influence on the friction coefficient of the parts in contact.

The initial linear contact between the disc and a block was adopted, which after the elastic and plastic deformation of elements, became a surface contact. There, the unit contact pressure from the extremely high value at the beginning of the contact, dropped to the level of measurable—operating pressure.

In these investigations, the sliding speed in the tribological pair block-disc was 2.93 m/s. The maximum sliding speed was calculated according to a well-known formula, where the kinematic radii of the examined gears are equal (129 mm) and their angular speeds are equal as well (850 min^−1^). The duration was selected according to some previous tests conducted in the laboratory. The tests were performed according to ASTM G99-17 standard.

It is known that the friction coefficient is not linearly dependent on the load and that it significantly increases at very high pressures. During these tribological tests, the normal (working) force was kept constant for the whole duration of the tests.

Lubrication has the very important role in tribological processes. In these tests the influence of various lubricating agents on the friction coefficient values was not investigated; the oil HIPOL B SAE 90 was used for lubrication.

Investigation of the tribological properties of the hard-faced blocks and cemented discs was done on the TPD-93 tribometer [3]. The disc rotates with angular velocity of ω = 92.7 s^−1^, and the block is fixed, firmly placed in the specially constructed holder. Lubrication was done by capturing a certain amount of oil using the lower part of the disc that was immersed in it, (Figure 1c). Lubrication was limiting. The line contact was realized by touching of the block’s frontal surface and the circumferential surface of the disc. The blocks marking is shown in Table 6. Discs were cemented and heat treated after the machining and has the same notation as their respective contacting blocks.

The primary goal of the tribological investigations was to determine the wear resistance of the deposited hard-faced layers and the base metal. That is why the contact block-on-disc was executed, where the variable parameters were only the tested materials. In other words, the wear parameters were in the focus of investigations (the friction coefficient and wear trace) of the blocks reparatory hard-faced with various filler metals. For that reason, the fixed parameters in these tribological tests were

- The contact force—F = 250 N,
- The sliding speed—v_sl_ = 2.93 m/s,
- Lubricant-Limiting (oil HIPOL B SAE 90),
- The contact duration—t = 30 min.

At the same time, the following technological parameters were variable:

- Type of the filler metal,
- Technological procedure execution (hard-facing prior to or after the heat treatment),
- Heat treatment regime.

**Figure 17:**
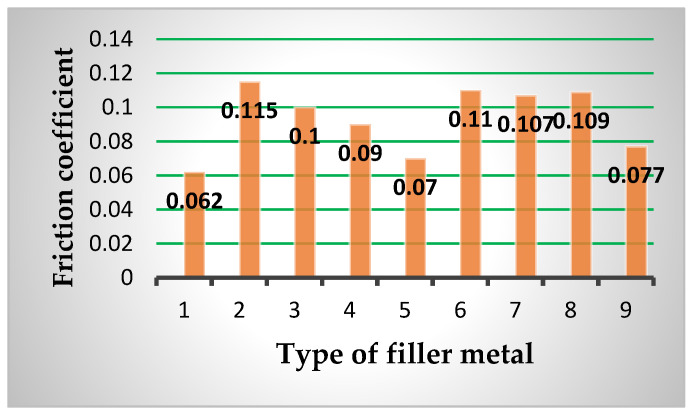
Comparison of the average values of the friction coefficient for various filler metals (Numbers correspond to notation of Table 7).

**Figure 18:**
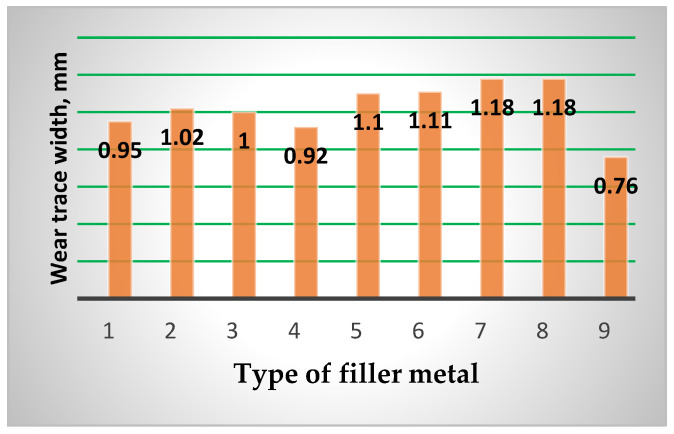
Comparison of the wear traces values for various filler metals (Numbers correspond to notation of Table 7).

Based on the conducted tribological investigations, the average friction coefficient values were determined (μ_av_), which are shown in Table 7 and in Figure 17. Topography of surfaces of the tested blocks was determined as well. The wear trace width was measured on a universal microscope UIM-21 and the results are presented in Figure 18.

Analyzing the friction coefficient determination results of the listed filler metals, one can conclude that the lowest values were obtained for the hard-facing executed by combination of the FMs Inox 18/8/6 and EDUR 600. Besides that, the FM Tooldur provides the value of the friction coefficient lower than the cemented and quenched blocks (of course, all in the contact with the cemented and quenched discs made of 20MnCr5 steel). The largest values of the friction coefficient were exhibited by the blocks hard-faced with FM Castolin 2, then the hard-faced layers deposited by the “soft” filler metals (EVBCrMo, Phönix 120 K/E 425 B/E7018-1 and EVB2CrMo), which were subsequently cemented and heat treated.

**Table 7:** Friction coefficients (μ_av_) and wear traces values for various filler metals.

Disc		Block			Friction Coefficient μ_av_	Wear Trace (mm)
Material	Hardness HRC	Sample No.	Filler Metal	Welding Procedure		
20MnCr5	55–58	1	Inox 18/8/6 + EDUR 600	MMAW (111)	0.064	0.960
		2	Castolin 2		0.115	1.028
		3	DUR 600-IG	GTAW (TIG) (141)	0.100	1.020
		4	UTP 670	MMAW (111)	0.090	0.955
		5	Tooldur		0.072	1.118
		6	EVBCrMo		0.110	1.130
		7	EVB2CrMo		0.106	1.198
		8	Phönix 120 K/E 425 B/E7018-1		0.108	1.200
		9	20MnCr5	/	0.077	0.751

What concerns the wear traces (what is of the utmost importance) is the elements that were cemented and quenched had the smallest values by far. From the hard-faced blocks the best appearance was by those obtained by the combination FMs Inox 18/8/6 and EDUR 600. The hard-faced layers obtained by the “soft” FMs Phönix 120 K/E 425 B/E7018-1 and EVB2CrMo were the worst from this aspect, as well.

## 6. Real Investigation of the Service Life of the Regenerated Gears

It must be emphasized here that the term “real” investigations assumes that they were conducted in conditions that are the closest to the operating conditions of the gear teeth surfaces. Investigations of the teeth flanks durability of the regenerated and newly manufactured gears were performed on a specially constructed device with the closed load circuit [19]. The calculated torque for the tested gears was T = 225 Nm at N = 51·10^6^ load cycles (1000 h of operation). To accelerate the tests, somewhat bigger torque value was applied T = 3000 Nm. That loading was not applied to gears instantly, but rather gradually, stepwise. In that way, the possible breaks during the running-in period were avoided. The newly made gears and the hard-faced ones were subjected to this test.

Appearance of the destructive wear (namely the first pits) was noticed after 68·10^6^ load cycles on teeth hard-faced by the filler metals Castolin 2 and EVBCrMo. On the newly made teeth, the pits started to appear at 69·10^6^ load cycles. The longest service life up to the first pits appearance had teeth hard-faced with the filler metal DUR 600-IG; the period of destructive pitting started after 74·10^6^ load cycles.

**Figure 19:**
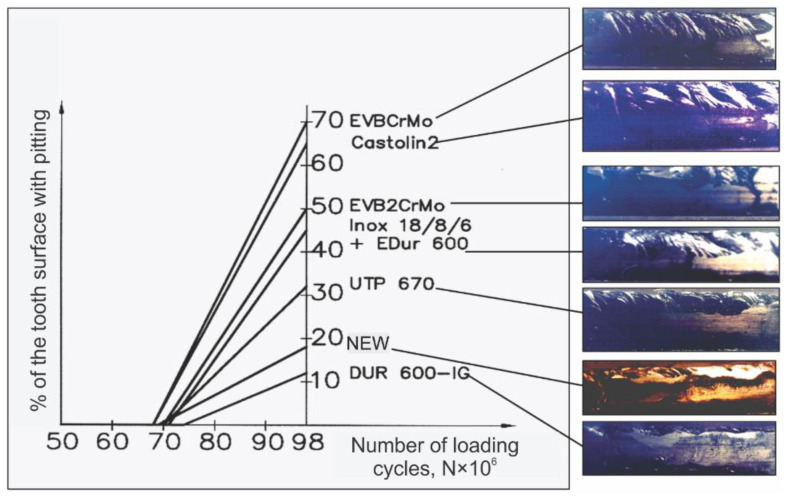
The development rate of the destructive pitting on the teeth flanks.

Analyzing the periods of the service life until appearance of the destructive pitting, of the regenerated and newly made gears, led to a diagram shown in Figure 19. When drawing this diagram, the dependence of the destructive pitting rate, namely increase of the percentage share of the teeth area, affected by destructive pitting, on the load cycles number (i.e., operating time of the testing device) was assumed as a linear function. This dependence is not linear, however, for the purpose of illustrating its trend, it is presented as a straight line whose slope determines the spreading rate of the pits that define the destructive pitting.

What concerns the phenomenon of pitting, it is noticed that the threshold exists when the first pits appear, for all the tested gears, around 70·10^6^ load cycles, as well as that all the applied hard-facing procedures produce the effect similar to the newly made gears. However, in the following phases of the destructive pitting, one can notice significant differences in its development, what is obvious from the diagram shown in Figure 19. The best results are exhibited by the new gears and those regenerated by hard-facing with DUR 600-IG filler metal.

In the initial period of the test, slightly greater wear was on the pinion leg near the base circle. The least wear was in the area of the base circle of the gear, even though the teeth on that part were in a single mash, but their sliding speed was the lowest. In the period of normal wear, it is noticed that the wear of the tooth surfaces per unit of time is very small. The removal of metal from the contact surfaces of the teeth (both reparative welded and cemented) during the break-in period and especially during the period of normal wear, was extremely small. The wear and tear were so small that it could hardly be felt by the touch of the finger, but was visually observed, that is, by the change in the color of the parts of the teeth that were in contact, compared to the color of the parts of the teeth that did not engage.

Authors believe that the gradual increase in the load during the break-in period contributed to a significantly lower rate of wear during the break-in and normal wear periods and probably to a significant increase in the service life of all the tested gears, as well. After all, that is the task of pairing (running in) heavily loaded gears. The biggest wear was in the leg of the tooth, i.e., under the base circle. The reasons for this are known: that is the zone of the beginning of the profile single connection, when the sliding speeds are higher and the influence of stress concentration, caused by the shape and geometry of the teeth, must not be ignored. The wear of the tooth heads was slightly less. Considering the length of the normal wear period, one got the impression that with this intensity of wear of the working surfaces of the teeth, the gears could work almost indefinitely. However, material fatigue occurs over time. The mechanism of fatigue wear here is also consistent with the one explained in literature thus far.

The specificity of the pitting development of repaired teeth is represented by the fact that the greatest destruction of the material occurred in the area where the junction of the two welding passes was located. Teeth welded by the TIG process, with filler metal DUR 600-IG, had the slowest development of destructive pitting due to the welding technique itself (the weld is applied by oscillatory movement of the wire over the entire height of the tooth).

From the aspect of durability of the sides of the teeth, i.e., the resistance to fatigue wear, the best characteristics are provided by the teeth repaired by the TIG process with the filler metal DUR 600-IG. The teeth welded with the filler metal Castolin 2 performed the worst, as well as the teeth welded with the “soft” filler metals EVBCrMo and EVB2CrMo, which were subsequently cemented and heat treated. However, their working life was also longer than expected.

Common among all the welded gears is the invariability of their microstructure before and after the tribological tests of the gears. However, the initial cracks were observed in some samples, which is the hint of the destructive pitting development. The cracks originated in the surface layer, thus they spread radially into the subsurface layer of the teeth, merging and forming pits. Residual stresses from welding, concentrated under the surface of the welded teeth and the inhomogeneity of the structure, as a result of the introduction of heat during welding, significantly help the development of fatigue wear, i.e., significantly determine the speed of development of the destructive pitting.

From analysis of the hard-faced layers, executed with the “soft” filler metals (EVBCrMo and EVB2CrMo), one can conclude that the significantly faster development of the destructive pitting for these teeth is a result of the chemical composition inhomogeneity of the teeth sub-surface layers, unfavorable microhardness distribution along the teeth cross-section, lower microhardness levels in the sub-surface layers (especially in the cemented zone), presence of the bigger share of the residual austenite, which represents the soft phase, namely it is the poor heat conductor.

The worse parameters of the teeth flanks durability, reparatory hard-faced with the “hard” filler metals (Castolin 2 and UTP 670), namely the faster development of the destructive pitting, could be a consequence of the fact that those materials (primarily Castolin 2) are aimed for regeneration of tools and not the construction machine parts. Their chemical composition is determined based on that, thus the properties of the hard-faced layers, as well. That is illustrated by inhomogeneity of otherwise fine dendrites in the microstructure of the hard-faced layer obtained with filler metal Castolin 2.

## 7. Conclusions

Based on the conducted tribological tests, the conclusion was reached that, from the aspect of the wear resistance (the smallest wear trace width), the most favorable is the base material (20MnCr5), then follows the hard-faced layer with the filler metals UTP 670. From the energy aspect (minimum friction coefficient), the hard-faced layer obtained with filler metals Inox 18/8/6 + EDUR 600 and Tooldur are even better than the base metal.

By analyzing the results obtained by testing gears regenerated by reparative hard-facing, it was possible to draw the following conclusions:

- geometric characteristics, prescribed tolerances and deviations, as well as the quality of surface treatment of regenerated gears, completely match the prescribed and standardized values of newly manufactured gears;
- the required mechanical characteristics, primarily the surface hardness, are easily obtained by applying appropriate filler materials and the heat treatment prior to and after the hard-facing;
- the improvement of all the mechanical characteristics of regenerated gears can be achieved by careful and well-organized preparation for hard-facing, selection of adequate filler materials and welding regimes, and strict adherence to prescribed operating modes, as well as precisely defined and carefully performed procedures of the heat treatment of the teeth working surfaces;
- bearing in mind the chemical compositions of the basic and filler materials, as well as their micro- and macrostructure, it can be concluded that the flanks of the welded teeth, provided that quality welding and machining are ensured, have hardness and wear resistance almost the same as the newly manufactured teeth;
- since the electrodes were used for welding, characterized by the low heat input in the coating application zone, it was metallographically confirmed that the shape of the welded structure is quite favorable;
- microhardness analysis shows that the welds made with “hard” filler materials have a higher microhardness than the cemented and hardened base metals, while the situation is reversed for the welds executed by the “soft” filler materials;
- The subject of selecting the optimal regime for the chemical-heat treatment can be a topic of separate research.

Based on analysis of the micro-structure, one can assume the following:

- Considering the wear resistance, one should expect the better results for the hard-faced layers executed by the “hard” filler metals and the newly made samples,
- Considering the fracture resistance, the best characteristics should be exhibited by the newly made samples.

Metallographic analysis in the hard-faced layers and HAZ has shown that neither hot nor cold cracks were detected, which usually appear as a consequence of the hard-facing and the subsequent heat treatment. In addition, the presence of undercuts, slag inclusions, etc. were not noticed. The presence of individual gas pores in the joint zone of the weld metal with the base material was determined in some hard-faced samples. However, those cannot significantly affect the exploitation behavior of these layers.

One of the main objectives of the conducted experiments was to select the base metal and the filler metals for the reparatory hard-facing of the meshed teeth. Since these are the heavily loaded gears, the material chosen for their manufacturing was steel 20MnCr5. The hardness required had to be greater than 55 HRC. All the discs were made of this steel and possessed the required hardness. The blocks, also made of the same material, were hard-faced in two principally different ways, and they also had the required hardness.

Hardness is definitely one of the most influential factors regarding the wear resistance properties of surfaces in contact. That is the reason of the presented selection of the filler metals for the hard-facing: those are the filler metals that produce the high surface hardness and/or the filler metals that would enable for their hardness to be raised to the required level by adequate post heat treatment.

## Figures and Tables

**Table 1:** Chemical composition of the steel used for samples.

Alloying Elements (%)					
C	Si	Mn	P_max_	S_max_	Cr
0.17–0.22	0.15–0.40	1.1–1.4	0.035	0.035	1.0–1.3

**Table 6:** Experimental blocks notation and hard-facing procedure.

Block No.	Welding Procedure	Filler Metal
1	MMAW	Inox 18/8/6 + EDUR 600
2		Castolin 2
3	GTAW	DUR 600-IG
4	MMAW	UTP 670
5		Tooldur
6		EVBCrMo + C + HT
7		EVB2CrMo + C + HT
8		Phönix 120 K/E 425 B/E7018-1 + C + HT
9		C + HT without hard-facing

## Data Availability

Not applicable.
